# Perceptions of the educational environment among undergraduate physical therapy students in a competency-based curriculum at the University of Chile

**DOI:** 10.3352/jeehp.2019.16.9

**Published:** 2019-04-29

**Authors:** Pablo Quiroga-Marabolí, Marcela Andrea Antúnez-Riveros, Marcela Aguirre-Jerez, Alvaro Besoain Saldaña, José Peralta-Camposano, María Pilar Ruiz de Gauna Bahillo

**Affiliations:** 1 Department of Physical Therapy, Faculty of Medicine, University of Chile, Santiago, Chile; 2 Department of Health Sciences Education, Faculty of Medicine, University of Chile, Santiago, Chile; 3 Laboratory for Scientific Image Analysis, Center for Medical Informatics and Telemedicine Program of Anatomy and Developmental Biology, Biomedical Science Institute, Faculty of Medicine, University of Chile, Santiago, Chile; 4 School of Medicine, Faculty of Medicine, University of Chile, Santiago, Chile; 5 Department of Theory and History of Education, Faculty of Philosophy and Educational Sciences, Basque Country University, UPV/EHU, Bilbao, Spain; Hallym University, Korea

**Keywords:** Competency-based education, Dundee Ready Education Environment Measure questionnaire, Education environment, Chile

## Abstract

**Purpose:**

This study aimed to assess the educational environment (EE) among students in a physical therapy undergraduate program, to identify patterns in EE perceptions among the students by year, and to determine issues that should be addressed.

**Methods:**

The Dundee Ready Education Environment Measure (DREEM) questionnaire was used to explore the relationships among the total mean score, subscales, and items in a competency-based curriculum in the physical therapy program at the University of Chile. The DREEM questionnaire was filled out by 166 of 244 students (68.03%), of whom 56.6% were men and 43.4% were women, with 75.9% between 19 and 23 years of age.

**Results:**

The total mean score (120.9/200) indicated that the EE was perceived as ‘more positive than negative.’ There were significant differences (P<0.05) between first-year students (113.41), who reported the lowest total mean score, and fourth-year students (126.60), who had the highest total mean score. Students rated their EE favorably on each subscale except social self-perceptions, which second-year students rated as ‘not too bad,’ and for which first-, third-, and fourth-year students gave a rating corresponding to ‘not a nice place.’ On the perceptions of teachers subscale, there were significant differences (P<0.05) between first-year students (28.05/44) and fourth-year students (32.24/44) and between second-year students (28.72/44) and fourth-year students (32.24/44). On the academic self-perceptions subscale, there were significant differences (P<0.05) between first-year students (18.12/32) and second-year (21.68/32), third-year (22.33/32), and fourth-year students (21.87/32).

**Conclusion:**

Physical therapy students at the University of Chile had positive perceptions of their EE. First-year students rated the largest number of items as problematic. Improvements are required across the program in the specific subscales mentioned above.

## Introduction

The educational environment (EE) includes all the variables (academic, social, and organizational) that interact to influence students’ personal and academic well-being [1,2]. The EE is an intricate web of emotional, intellectual, and physical strings that are socially constructed by individuals [2], and it affects academic performance, quality of life, attitude towards scholarly activities, and students’ ability to handle the stress that is inherent in health educational programs [1].

The EE also involves the relationships among subjects that play a role in the educational process in both formal and informal spaces and become a part of educational institutions’ good practice [2]. Students are exposed to a new social and EE in a particular curricular context that could be measured to identify areas for improvement. Measures of EE could help to improve a learner-centered curriculum that involves faculty and students and facilitates opportunities for achieving learning outcomes and promoting professional development [3,4].

Competency-based education covers a series of changes that require an analysis not only of the contents, strategies, and evaluation of teaching-learning processes, but also aspects related to educational institutions, the curriculum, and the culture of teachers and students [5], issues which could be addressed by EE assessments.

The University of Chile is a public educational institution consisting of numerous faculties; student entrance is administered through a university selection test, and there are 3 sources of financing for students: tuition fees, student loans, and fiscal transfer. Since 2006, it has been committed to a competency-based model with the Board of Rectors of Chilean Universities beginning the process of curriculum innovation, reflected in the institutional development plan. Its progression requires the consideration of epistemological, ontological, and didactic dimensions, enabling a transition towards an integrated model that includes an early approach in the health professions programs of the faculty of medicine to real professional practice and visualizing the complexity of health actions in educational programs [6].

The physical therapy school in the faculty of medicine at the University of Chile has been a pioneer in the process of curriculum innovation. In 2009 the physical therapy school was the first to implement a competency-based curriculum covering 5 domains of competencies: health and study of human movement as a core domain, research, public health management, education, and a generic transverse domain common to all health professions of the faculty of medicine.

To date, however, no data have been collected to evaluate the EE after implementation of this new curriculum, and no information is available about the EE in the older curriculum.

The new physical therapy program provides 60 credits per year (1 credit=27 hours). During the first 2 years, the focus is on basic science and pathophysiology, with teaching and learning strategies including lectures, small-group sessions, and problem-based learning on simple contexts. Simulation-based learning experiences and clinical learning activities in the areas of respiratory care, musculoskeletal rehabilitation, and neurological rehabilitation begin in the second year of study to introduce progressive practical clinical skills, with a focus on problem-based approach. Fourth-year students integrate their competencies in courses of professional intervention in context using clinical reasoning strategies. Fifth-year students must complete a clinical internship.

Soemantri suggested that the Dundee Ready Education Environment Measure (DREEM) is the best questionnaire for evaluating the EE in undergraduate health education programs, as it has been shown to have good reliability and validity in a variety of cultures and contexts. The DREEM can be used for many purposes, including the identification of potential problem areas, comparison of different groups, comparison of different conditions in the same group, and examinations of the relationship of the EE with other measures [7]. The DREEM has been validated for use in many languages and countries [8,9], including in Chile [10-12], with good reliability and internal consistency in multiple learning contexts.

This study aimed to assess perceptions of the EE among students in a physical therapy undergraduate program; to identify patterns in their perceptions of the EE by year in the program, and to determine issues in the EE that should be addressed.

## Methods

### Ethics statement

This study was approved by the Ethics Committee of the Faculty of Medicine, University of Chile (protocol project no., 008-2017). Only those who signed the informed consent form were given questionnaires.

### Study design

A cross-sectional survey research design was used.

### Materials and subjects

This research used a non-probabilistic convenience sample. The participants were students of the University of Chile physical therapy program in Santiago, Chile. A total of 244 students were invited to participate during the second academic semester of 2016. The inclusion criteria were regular students from first to fourth year of the program. Fifth-year students were excluded because they were participating in their clinical internships. Ultimately, 166 students signed the informed consent form and completed the questionnaire in a single day on which obligatory university activities had to be carried out, through prior coordination with the instructors of the program, who verbally explained the objective and scope of the investigation. The sample enrolled represented 68.03% of the physical therapy program. The use of the DREEM questionnaire from Roff et al. [13] was permitted by Professor Sue Roff.

The Spanish version of the DREEM questionnaire validated by Riquelme et al. [10] consists of 50 Likert-type items with 5 levels: 4, strongly agree; 3, agree; 2, unsure; 1, disagree; and 0, strongly disagree. Negative items are scored in reverse, so that a higher score always indicates a more positive evaluation. Results are tabulated for total mean score and for each subscale. Use of the Spanish version of DREEM questionnaire was permitted by Dr. Riquelme [10].

The total mean score has a maximum of 200 points, interpreted as follows: 0–50 points, very poor; 51–100 points, significant problems; 101–150 points, more positive than negative; and 151–200 points, excellent. The 5 subscales are interpreted as follows: students’ perception of learning (12 items, maximum score=48), students’ perceptions of teachers (11 items, maximum score=44), students’ academic self-perceptions (8 items, maximum score=32), students’ perception of atmosphere (12 items, maximum score=48) and students’ social self-perceptions (7 items, maximum score=28).

To identify problem areas or issues that need attention, each item is analyzed separately according to its average score [13]. Items with mean scores ≤2 indicate problematic areas, scores between 2 and 3 indicate areas that could be enhanced or improved, and scores ≥3.5 represent strong areas.

### Statistics

Descriptive and inferential analysis was performed using IBM SPSS ver. 23.0 (IBM Corp., Armonk, NY, USA). The total mean score, subscales, and item scores were tabulated and analyzed according to the methods suggested by Roff et al. [13] for the total sample and by year of study. Negative items (items 4, 8, 9, 17, 25, 35, 39, 48, and 50) were scored in reverse so that a higher score always indicated a more positive evaluation. The total mean score and subscale scores were tabulated as a whole and by year of study. The individual items were analyzed according to the methods suggested by Swift et al. [14]. The Shapiro-Wilk test was used to evaluate the normality of the data. Analysis of variance (ANOVA) was used to determine the significance of differences among years of study. The Bonferroni correction was used to adjust for multiple comparisons. The level of significance was set at P<0.05. Descriptive analysis was performed to calculate the mean score for each item; these item scores were then used to identify problem areas as a whole or by year of study.

The Cronbach α was used to evaluate the internal consistency of the DREEM results, for both the total mean and subscale scores. The Cronbach α was 0.919 for the total mean score, 0.798 for students’ perception of learning, 0.769 for students’ perceptions of teachers, 0.693 for students’ academic self-perceptions, 0.757 for students’ perceptions of atmosphere, and 0.611 for students’ social self-perceptions. The sample characteristics are shown in Table 1.

Following the method recommended by Swift et al. [14], the Shapiro-Wilk test (P>0.05) was used to confirm the normality of the distribution of the overall and subscale score data. The t-test was then used to evaluate significant differences by gender and year of study. The raw data are available in Supplement 1.

## Results

### Total mean and subscale scores

The total mean score of the sample was 120.9 (standard deviation [SD]=21.3). The total mean scores by year of study were 113.41 (SD=23.4) for first-year students, 121.46 (SD=19.8) for second-year students; 121.46 (SD=22.7) for third-year students, and 126.6 (SD=18.6) for fourth-year students. The first-year students gave the lowest overall rating for the EE, and the fourth-year students gave the highest rating.

Table 2 shows the mean subscale and total mean DREEM scores. Students in all years of study rated the EE overall as ‘more positive than negative,’ and also provided similar subscale scores for 4 of the 5 dimensions evaluated. Perception of learning was rated as ‘a more positive approach,’ perceptions of teachers as ‘moving in the right direction,’ academic self-perceptions as ‘feeling more on the positive side,’ and perceptions of the atmosphere as ‘a more positive atmosphere.’ Second-year students rated social self-perceptions as ‘not too bad,’ while first-, third-, and fourth-year students rated this dimension of the EE as ‘not a nice place.’ The first-year students gave the lowest ratings for all subscales except social self-perceptions (Table 2).

When comparing scores by year of study using ANOVA, significant differences (P<0.05) were found for the total mean score (P=0.049), perceptions of teachers (P=0.0001), and academic self-perceptions (P=0.001). Using the Bonferroni correction for multiple comparisons, it was found that the difference between first- and fourth-year students (P=0.024) in the total mean score was statistically significant. For the perceptions of teachers subscale, the differences between first- and fourth-year students (P=0.00057) and between second- and fourth-year students (P=0.0032) were statistically significant. For the academic self-perceptions subscale, the differences between first- and second-year students (P=0.0007), first- and third-year students (P=0.013), and first- and fourth-year students (P=0.00046) were significant (Table 2).

### Individual item scores

As recommended by Swift et al. [14], individual items were evaluated to identify problems in the EE, with items with a mean score ≤2 needing particular attention (Table 3).

The total mean score for the total sample revealed a total of 11 items from 3 subscales that were potential problem areas (items 3, 4, 9, 12, 14, 17, 25, 27, 29, 42, and 46). The items identified as problematic by students in all years of study were: “I am able to memorize all I need” (academic self-perceptions), “the course is well timetabled” (perceptions of atmosphere), “cheating is a problem in this course” (perceptions of atmosphere), “the enjoyment outweighs the stress of the program” (perceptions of atmosphere), “there is a good support system for students who get stressed” (social self-perceptions), and “I am too tired to enjoy this course” (social self-perceptions).

First-year students rated the largest number of items as potential problem areas (with 17 items from all 5 subscales) (items 3, 4, 5, 9, 12, 14, 17, 22, 25, 26, 27, 29, 30, 42, 44, 47, and 49). Second-year students rated 13 items as potential problem areas from all 5 subscales (items 3, 4, 9, 12, 14, 17, 25, 27, 29, 42, 44, 46, and 48); third-year students 12 items from all 5 subscales (items 3, 4, 9, 12, 14, 17, 25, 27, 29, 42, 46, and 48) and fourth-year students 9 items (items 3, 4, 12, 14, 17, 22, 27, 42, and 46) from the subscales of academic self-perceptions, perception of atmosphere, and social self-perception. There were no significant gender differences in the analyzed sample.

## Discussion

The response rate of 68%, the analytical methods used in this study, and the gender ratio of the sample are comparable to other studies with similar sample sizes [2,12,15,16].

The rating of the EE as ‘more positive than negative’ found in this sample is similar to what has been reported in other studies evaluating physical therapy programs [2,4,12,17,18]; however, we obtained lower values for the total mean score (120.9), which could have been due to the low scores of the perception of learning and social perceptions subscales affecting the total mean score, similar to the findings of Odole et al. [4]. These findings are similar to the scores obtained by Sunkad et al. [17], who evaluated health care programs and found that physical therapy had the lowest scores. Sunkad et al. [17] also found that undergraduate students had lower scores than postgraduates.

The first-year students reported the lowest ratings out of the entire study sample. This finding may have been due to the fact that first-year students enter directly from secondary school, meaning that they were likely experiencing differences in many domains (such cognitive domain and scientific background) when compared with the students in subsequent years of the program [3]. Furthermore, high expectations regarding the EE may not be met during the first semester, resulting in a drop in satisfaction when students learn more about the reality of university life, the teaching strategies used, and the volume of work required. As students learn more about their area of interest, they may develop more capacity for critical reflection [3].

The higher total mean score reported by from students in the third and fourth years may have been due to the imminence of professional development [2,3], as well as their adaptations to the clinical learning environment and their knowledge of the core curriculum content of the physical therapy program [3].

In terms of perceptions regarding instructors and teaching methods, students in the first and third years reported that the teachers used an authoritarian approach, provided insufficient feedback, and over-emphasized memorization. This finding may be explained by the fact that students in the first year of this physical therapy program are tasked with mastering large volumes of information, potentially creating stress, especially when the students do not perceive a clear link between the information and their future practice [2,19]. Moreover, first-year students take a large proportion of basic science courses, making it more difficult for students to link their learning with their first clinical activities [12]. It is also possible that despite the implementation of a learner-centered and competency-based curriculum, some teachers may be resistant to change, may continue to follow traditional ways of teaching, and may be uncomfortable with their new roles [2,5,19]. In the fourth year of the program, students experience a variety of learning strategies and engage more deeply in clinical work in different areas of physical therapy practice [12], such as respiratory care, musculoskeletal rehabilitation, and neurological rehabilitation, which draw upon skills they have developed in the program. This could generate a better perception of the EE due to their proximity with real professional life [3,12].

Perceptions of inadequate support for helping students to manage stress and fatigue are a common problem in health education programs [2,16]. Health sciences students may face many issues, including personal problems (relationship issues, lack of time to pursue recreational activities), professional problems (issues with the structure of the university or relationships with teachers), academic problems (low academic performance) and administrative problems (issues with curricular content or length). If these issues are not adequately addressed, students’ well-being and academic performance could be affected. Unfortunately, innovative curriculum models have yet to overcome these issues [16]. Academic demands are often perceived as greatest during the first years of the program. Failure to adequately explain the learning objectives and purpose of each course may exacerbate feelings of stress and anxiety [16]. The relatively low ratings for the perceptions of teachers, perceptions of atmosphere, academic self-perceptions, and social self-perceptions in this study may be attributable to the mentioned factors.

The problem areas identified in this study are similar to those found in other physical therapy programs, including a teaching-centered approach that overemphasizes memorization, stress or fatigue due an overloaded curriculum and lack of information about mental health support, and cheating in the program due an overloaded curriculum [2,4,10,12,18], and these factors must be taken into account in the evaluation of the new curriculum of the program.

**Fig. 1.**
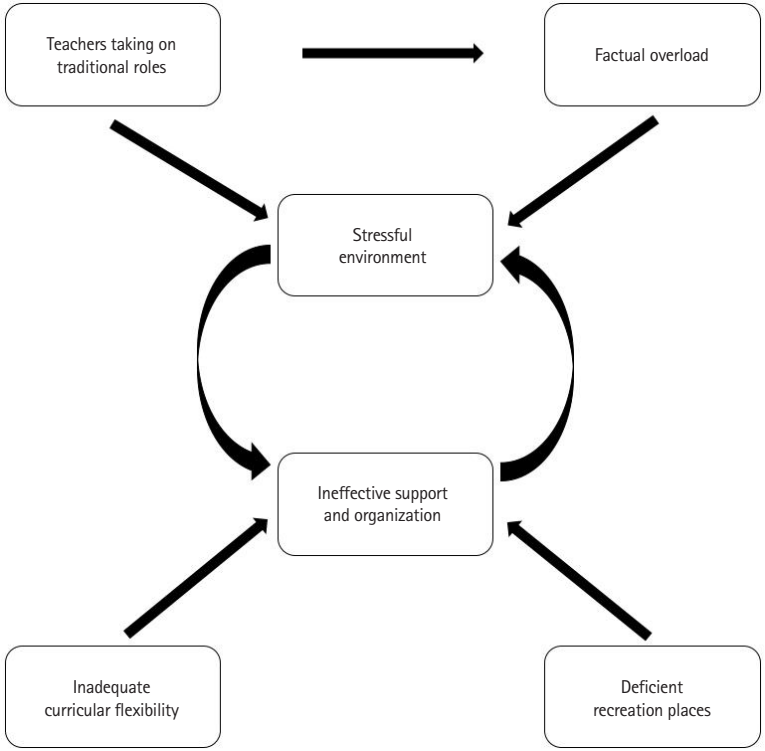
Adaptation of the model proposed by Palmgren et al. [2] in 2015 to illustrate the relationships among problem areas identified in the study. Modified from Palmgren et al. J Chiropr Educ 2015;29:110-126 [2].

Finally, we propose utilizing a model adapted from Palmgren et al. [2] to improve institutional support, due to its simple interpretation, clear empirical evidence, straightforward theoretical framework, and similarity with the context of the physical therapy program of the University of Chile (Fig. 1). This adapted model could be used to illustrate the relationships among the problem areas identified in this particular study, revealing a cycle between the stressful environment and lack of effective support to help students to manage stress in the program, while the perceived demands of the program are exacerbated by traditional teaching methods (inadequate feedback and a teacher-centered approach), inflexibility within the curriculum, and lack of spaces for recreation and leisure. This proposed model could help to understand the EE, taking into account the most common factors that should be addressed according to the problems detected in particular items and years of the program, and could also improve conditions in a way that promotes the proper development of the EE.

Students’ evaluations of the EE proposed in this research are only applicable to the time frame when the data were gathered. The exclusion of fifth-year students due to their clinical activities meant that it was not possible to obtain an overview of the totality of the students in the program.

In conclusion, the students perceived a good EE in general terms in the period during which the evaluation was conducted. First-year students perceived their EE as more adverse, presenting the lowest values in perception of learning, perceptions of teachers, academic self-perceptions, and perceptions of the atmosphere. The main problems in first-year students were linked to problems with teachers, teaching methods, perceptions of academic load, cheating, fatigue, and lack of support in situations of stress. These findings have been used to improve teaching-learning processes and to analyze the problems of the EE in the current 2014–2018 curricular evaluation process. This information will be used in the next round of curricular planning in the 2019–2023 physical therapy development plan. It is recommended to complement the findings of this research with a qualitative study to explore the current context and to propose potential solutions to the problematic items.

## Figures and Tables

**Table 1.** Age and gender of students, by year of study and for the total sample

Year of study	Total	Gender		Age (yr)		
		Male	Female	18	19–23	≥24
I	41 (24.7)	23 (56.1)	18 (43.9)	9 (22.0)	29 (70.7)	3 (7.3)
II	50 (30.1)	30 (60.0)	20 (40.0)	0	49 (98.0)	1 (2.0)
III	30 (18.1)	18 (36.0)	12 (24.0)	0	19 (63.3)	11 (36.7)
IV	45 (27.1)	23 (51.1)	22 (48.9)	0	29 (64.4)	16 (35.6)
Total (I–IV)	166 (100.0)	94 (56.6)	72 (43.4)	9 (5.4)	126 (75.9)	31 (18.7)

Values are presented as number (%).

**Table 2.** Mean total and subscale DREEM scores of physical therapy students from the University of Chile

DREEM subscale	Max score	All years (N=166)		First year (n=41)		Second year (n=50)		Third year (n=30)		Fourth year (n=45)		Significant differences by year level (P<0.05)^b)^
		Score±SD	% Max	Score±SD	% Max	Score±SD	% Max	Score±SD	% Max	Score±SD	% Max	
Total mean score^a)^	200	120.9±21.3	60.44	113.41^c)^±23.4	56.7	121.46^c)^±19.8	60.73	121.63^c)^±22.7	60.82	126.60^c)^±18.6	63.3	1:4
Perception of learning	48	27.8±6.3	57.97	26.46^d)^±7.2	55.13	27.86^d)^±5.8	58.04	28.00^d)^±6.8	58.33	28.91^d)^±5.7	60.23	NA
Perceptions of teachers^a)^	44	29.62^e)^±5.1	67.32	28.05^e)^±5.0	63.75	28.72^e)^±4.6	65.27	29.33^e)^±5.8	66.67	32.24^e)^±4.3	73.28	1:4, 2:4.
Academic self-perceptions^a)^	32	20.79^f)^±4.5	64.97	18.12^f)^±4.8	56.63	21.68^f)^±4.2	67.75	21.33^f)^±3.7	66.67	21.87^f)^±4.1	68.33	1:2, 1:3, 1:4
Perceptions of atmosphere	48	28.0^g)^±6.2	58.38	26.27^g)^±6.8	54.73	28.00^g)^±6.3	58.33	28.43^g)^±6.1	59.24	29.38^g)^±5.5	61.2	NA
Social self-perceptions	28	14.6^h)^±3.9	52.28	14.51^h)^±3.9	51.83	15.2^i)^±4.3	54.29	14.53^h)^±3.7	51.9	14.20^h)^±3.5	50.71	NA

Values are presented as mean±SD or % Max. DREEM, Dundee Ready Education Environment Measure; SD, standard deviation; NA, not applicable.

a) Statistically significant analysis of variance result.

b) Bonferroni-corrected for multiple comparisons. Interpretation:

c) more positive than negative;

d) a more positive approach;

e) moving in the right direction;

f) feeling more on the positive side;

g) a more positive atmosphere;

h) not a nice place; and

i) not too bad.

**Table 3.** Mean scores of physical therapy students from University of Chile for DREEM items

DREEM items	Total sample	First year	Second year	Third year	Fourth year
1. I am encouraged to participate in class. ^SPLSp^	2.6±0.9	2.5±0.8	2.6±1.0	2.8±0.8	2.6±0.8
2. The teachers are knowledgeable. ^SPTS^	3.6^a)^±0.5	3.7^a)^±0.5	3.6^a)^±0.5	3.6^a)^±0.6	3.6^a)^±0.5
3. There is a good support system for students who get stressed. ^SSSPS^	1.2^b)^±0.9	1.5^b)^±1.0	1.3^b)^±0.9	0.9^b)^±0.8	0.9^b)^±0.8
4. I am too tired to enjoy this course. ^SSSPS^	1.3^b)^±1.0	1.1^b)^±0.8	1.3^b)^±0.9	1.3^b)^±1.1	1.6^b)^±1.2
5. Learning strategies which worked for me before continue to work for me now. ^ASPSc^	2.1±1.2	1.6^1^±1.4	2.2±1.1	2.2±1.2	2.5±1.1
6. The teachers are patient with patients. ^SPTS^	3.0±0.8	2.6±0.8	3.0±0.9	3.3±0.5	3.2±0.8
7. The teaching is often stimulating. ^SPLSp^	2.5±0.9	2.3±0.9	2.5±0.9	2.5±0.8	2.5±0.9
8. The teachers ridicule the students. ^SPTS^	2.5±0.9	2.5±0.9	2.2±0.7	2.4±1.1	3.0±0.7
9. The teachers are authoritarian. ^SPTS^	1.7^b)^±1.0	1.5^b)^±1.0	1.4^b)^±0.9	1.4^b)^±1.0	2.3±0.9
10. I am confident about passing this year. ^ASPSc^	2.9±0.9	2.7±1.0	3.2±0.8	2.4±1.1	3.1±0.7
11. The atmosphere is relaxed during clinical teaching. ^SPASt^	2.6±0.9	2.8±0.8	2.7±0.9	2.6±1.0	2.4±1.0
12. The school is well timetabled. ^SPASt^	1.6^b)^±1.1	1.6^b)^±1.1	1.8^b)^±1.1	1.8^b)^±1.1	1.3^b)^±1.1
13. The teaching is student-centered. ^SPLSp^	2.4±1.0	2.4±1.1	2.3±0.9	2.3±1.1	2.5±0.9
14. I am rarely bored in this course. ^SSSPS^	1.6^b)^±1.0	1.2^b)^±0.9	1.7^b)^±1.0	2.0^b)^±1.1	1.6^b)^±0.9
15. I have good friends in this school. ^SSSPS^	3.3±0.8	3.5±0.6	3.4±0.8	3.3±1.0	3.0±0.9
16. The teaching helps to develop my competence. ^SPLSp^	3.1±0.8	3.0±0.9	3.1±0.7	3.3±0.6	3.1±0.7
17. Cheating is a problem in this school. ^SPASt^	1.4^b)^±1.2	1.5^b)^±1.2	1.1^b)^±1.0	1.6^b)^±1.4	1.6^b)^±1.3
18. The teachers have good communication skills with patients. ^SPTS^	3.2±0.7	2.7±0.9	3.4±0.6	3.4±0.6	3.3±0.5
19. My social life is good. ^SSSPS^	3.0±1.0	2.7±1.3	3.1±0.8	3.1±0.9	3.0±0.9
20. The teaching is well-focused. ^SPLSp^	2.6±0.9	2.5±1.0	2.7±1.0	2.7±0.9	2.7±0.7
21. I feel I am being well-prepared for my profession. ^ASPSc^	3.1±0.8	3.0±0.9	3.2±0.8	3.1±0.8	3.1±0.7
22. The teaching helps to develop my confidence. ^SPLSp^	2.1±1.0	2.0^b)^±1.1	2.4±0.9	2.3±1.1	2.0^b)^±1.0
23. The atmosphere is relaxed during lectures. ^SPASt^	2.7±0.9	2.5±0.9	2.7±0.8	2.4±1.2	3.0±0.7
24. The teaching time is put to good use. ^SPLSp^	2.3±1.0	2.5±1.1	2.4±1.0	2.4±1.1	2.2±0.9
25. The teaching overemphasizes factual learning. ^SPLSp^	1.5^b)^±1.0	1.1^b)^±0.9	1.3^b)^±0.9	1.7^b)^±1.0	2.1±0.9
26. Last year’s work has been good preparation for this year’s work. ^ASPSc^	2.7±1.1	1.7^1^±1.2	2.9±0.9	2.8±0.8	3.4±0.6
27. I am able to memorize all I need. ^ASPSc^	1.7^b)^±1.1	1.6^b)^±1.2	2.0^b)^±1.2	1.7^b)^±1.1	1.7^b)^±1.0
28. I seldom feel lonely. ^SSSPS^	2.6±1.1	2.5±1.2	2.6±1.0	2.7±1.1	2.6±0.9
29. The teachers are good at providing feedback to students. ^SPTS^	1.9^b)^±1.1	1.8^b)^±1.1	1.9^b)^±1.0	1.6^b)^±1.1	2.1±1.1
30. There are opportunities for me to develop interpersonal skills. ^SPASt^	2.3±1.1	1.9^b)^±1.1	2.3±1.2	2.4±1.1	2.5±0.9
31. I have learned a lot about empathy in my profession. ^ASPSc^	2.8±1.0	2.6±1.1	3.0±0.9	3.0±1.1	2.8±0.9
32. The teachers provide constructive criticism here. ^SPTS^	2.7±0.9	2.5±1.0	2.7±0.9	2.7±1.0	2.9±0.7
33. I feel comfortable in class socially. ^SPASt^	3.0±0.8	3.0±0.8	2.9±1.0	3.2±0.7	3.2±0.6
34. The atmosphere is relaxed during seminars/tutorials. ^SPASt^	2.7±0.9	2.7±1.0	2.5±0.9	2.6±1.1	2.8±0.7
35. I find the experience disappointing. ^SPASt^	2.8±0.9	2.7±0.9	2.9±0.8	2.7±1.1	2.9±0.9
36. I am able to concentrate well. ^SPASt^	2.2±1.1	2.1±1.2	2.2±1.0	2.2±1.2	2.3±1.2
37. The teachers give clear examples. ^SPTS^	2.8±0.8	2.7±0.8	2.8±0.7	2.7±0.8	3.1±0.7
38. I am clear about the learning objectives of the course. ^SPLSp^	2.5±1.0	2.5±0.8	2.5±0.8	2.1±1.3	2.7±1.0
39. The teachers get angry in class. ^SPTS^	2.7±0.9	2.5±0.9	2.5±0.8	2.5±0.9	3.2±0.6
40. The teachers are well prepared for their classes. ^SPTS^	3.3±0.7	3.4±0.7	3.3±0.7	3.4±0.8	3.4±0.6
41. My problem-solving skills are being well developed here. ^ASPSc^	2.4±0.9	2.2±1.0	2.4±0.9	2.8±0.8	2.4±0.8
42. The enjoyment outweighs the stress of studying Physical Therapy. ^SPASt^	1.6^b)^±1.1	1.3^b)^±1.1	1.8^b)^±1.0	1.7^b)^±1.4	1.8^b)^±1.1
43. The atmosphere motivates me as a learner. ^SPASt^	2.3±0.9	2.2±0.9	2.5±0.9	2.4±1.1	2.4±0.9
44. The teaching encourages me to be an active learner. ^SPLSp^	2.0±1.0	1.9^b)^±1.2	2.0^b)^±0.9	2.1±0.9	2.2±1.0
45. Much of what I have to learn seems to be relevant to a career in Physical Therapy. ^ASPSc^	2.9±0.9	2.8±1.1	2.9±1.0	3.4±0.6	2.9±0.9
46. My accommodation is pleasant. ^SSSPS^	1.7^b)^±1.2	2.1±1.2	1.8^b)^±1.2	1.2^b)^±1.2	1.5^b)^±1.0
47. Long-term learning is emphasized over short-term learning. ^SPLSp^	2.2±1.2	1.9^b)^±1.3	2.3±1.1	2.1±1.0	2.2±1.1
48. The teaching is too teacher-centered. ^SPLSp^	2.1±1.0	2.1±0.9	1.8^b)^±0.9	1.8^b)^±1.0	2.4±1.0
49. I feel able to ask the questions I want. ^SPASt^	2.7±1.0	2.0^b)^±1.1	2.6±1.0	2.8±1.0	3.2±0.7
50. The students irritate the teachers. ^SPTS^	2.2±1.0	2.2±1.0	2.1±0.9	2.3±1.1	2.4±1.0

Values are presented as mean±standard deviation. DREEM, Dundee Ready Education Environment Measure; ^SPLS^, items from the perception of learning subscale. ^SPTS^, items from the perceptions of teaching subscale. ^ASPSc^, items from the academic self-perceptions subscale. ^SPASt^, items from the perceptions of atmosphere subscale. ^SSSPS^, items from the social self-perceptions subscale.

a) Items with a mean score ≥3.5, particularly strong areas.

b) Items with a mean score ≤2, need particular attention.

## Supplementary materials

Supplement 1. Data files are available from https://doi.org/10.7910/DVN/ZRABRB.

Supplement 2. Audio recording of the abstract.
